# Montessori Method as a Cognitive Stimulation Tool in Patients Diagnosed with Major Neurocognitive Disorder: A Pilot Study

**DOI:** 10.1002/alz70857_101144

**Published:** 2025-12-25

**Authors:** Agustina Melnitzky, Alice Baez Lovera, Waleska Berrios, Maria Mercedes Garcia Basalo, Ivan Torrisi, Maria Cecilia Fernandez

**Affiliations:** ^1^ Hospital Italiano de Buenos Aires, Buenos Aires, Argentina; ^2^ Hospital Italiano de Buenos Aires, Buenos aires, Argentina; ^3^ Hospital ITaliano de BUenos Aires, Buenos Aires, Argentina

## Abstract

**Background:**

The Montessori Method (MM), adapted for individuals with Major Neurocognitive Disorder (MND) by Dr. Cameron (1990), uses cognitive stimulation (CS) principles such as task breakdown, repetition, and self‐assessment, combined with progressively challenging tasks. It also incorporates Tom Kitwood's (1997) Person‐Centered Care Model, focusing on the identity and interests of individuals with MND. MM encourages participation and promotes independence through meaningful activities.

In this study, MM was applied in group CS sessions to address language, memory, and orientation deficits. The intervention tailored activities to individual needs, emphasizing caregiver collaboration to continue CS at home.

The objective of this study is to explore the effects of CS sessions with MM on patients with MND and their caregivers, evaluating cognitive performance, mood, quality of life, patient functionality, and caregiver stress levels.

**Method:**

Ten patients with MND and their caregivers were assessed before and after three months of MM‐based CS sessions. Cognitive performance, mood, and quality of life were measured using the Mini‐Mental State Examination (MMSE), Hospital Anxiety and Depression Scale (HADS), and SF‐36 Health Questionnaire. Caregiver stress was evaluated with the Zarit Burden Interview and the Lawton and Brody Index.

**Result:**

The mean age of patients was 80.4 years, with an average of 11.3 years of education; 70% were female. After three months (average of 13.5 sessions), the MMSE score remained stable. Anxiety decreased by 1.4 points, while depression increased by 0.6 points. Functional status remained stable or improved in some patients. Mental health scores increased, but general health perception declined. Caregiver stress showed minimal change.

**Conclusion:**

After three months of MM‐based CS sessions, the sample showed stable cognitive performance and depression levels. A reduction in anxiety levels and an improvement in self‐perceived mental health were observed, suggesting that MM is effective in maintaining cognitive and emotional abilities in patients with MND. Caregivers also reported stable stress levels. While this is a pilot study and larger samples are needed to confirm these findings, preliminary results suggest that MM can be a valuable tool for cognitive and emotional intervention in patients with MND.

